# Deep resequencing identifies candidate functional genes in leprosy GWAS loci

**DOI:** 10.1371/journal.pntd.0010029

**Published:** 2021-12-08

**Authors:** Vinicius M. Fava, Monica Dallmann-Sauer, Marianna Orlova, Wilian Correa-Macedo, Nguyen Van Thuc, Vu Hong Thai, Alexandre Alcaïs, Laurent Abel, Aurélie Cobat, Erwin Schurr

**Affiliations:** 1 Program in Infectious Diseases and Immunity in Global Health, The Research Institute of the McGill University Health Centre, Montreal, Canada; 2 McGill International TB Centre, Montreal, Canada; 3 Department of Human Genetics, Faculty of Medicine and Health Sciences, McGill University, Montreal, Canada; 4 Department of Medicine, Faculty of Medicine and Health Sciences, McGill University, Montreal, Canada; 5 Department of Biochemistry, Faculty of Medicine and Health Sciences, McGill University, Montreal, Canada; 6 Hospital for Dermato-Venerology, Ho Chi Minh City, Vietnam; 7 Laboratory of Human Genetics of Infectious Diseases, Necker Branch, Institut National de la Santé et de la Recherche Médicale 1163, Paris, France; 8 Imagine Institute, Paris Descartes-Sorbonne Paris Cité University, Paris, France; 9 St. Giles Laboratory of Human Genetics of Infectious Diseases, Rockefeller Branch, Rockefeller University, New York, United States of America; Shandong Provincial Institute of Dermatology and Venereology, CHINA

## Abstract

Leprosy is the second most prevalent mycobacterial disease globally. Despite the existence of an effective therapy, leprosy incidence has consistently remained above 200,000 cases per year since 2010. Numerous host genetic factors have been identified for leprosy that contribute to the persistently high case numbers. In the past decade, genetic epidemiology approaches, including genome-wide association studies (GWAS), identified more than 30 loci contributing to leprosy susceptibility. However, GWAS loci commonly encompass multiple genes, which poses a challenge to define causal candidates for each locus. To address this problem, we hypothesized that genes contributing to leprosy susceptibility differ in their frequencies of rare protein-altering variants between cases and controls. Using deep resequencing we assessed protein-coding variants for 34 genes located in GWAS or linkage loci in 555 Vietnamese leprosy cases and 500 healthy controls. We observed 234 nonsynonymous mutations in the targeted genes. A significant depletion of protein-altering variants was detected for the *IL18R1* and *BCL10* genes in leprosy cases. The *IL18R1* gene is clustered with *IL18RAP* and *IL1RL1* in the leprosy GWAS locus on chromosome 2q12.1. Moreover, in a recent GWAS we identified an HLA-independent signal of association with leprosy on chromosome 6p21. Here, we report amino acid changes in the *CDSN* and *PSORS1C2* genes depleted in leprosy cases, indicating them as candidate genes in the chromosome 6p21 locus. Our results show that deep resequencing can identify leprosy candidate susceptibility genes that had been missed by classic linkage and association approaches.

## Author summary

Leprosy is an infectious disease caused by the *Mycobacterium leprae*. The development of an effective treatment in the 1980s contributed to the decline of leprosy prevalence. Still, around 200 thousand new leprosy cases are detected every year. In the past two decades, multiple studies have identified genetic variants increasing the risk of developing leprosy, particularly using genome-wide association studies (GWAS). However, GWAS loci commonly encompass multiple genes, which poses a challenge in identifying the best candidates contributing to susceptibility. This limits the usefulness of the genetic findings for understanding leprosy pathogenesis. Our study tested if the accumulation of rare protein-altering variants in genes located in GWAS loci could distinguish the best functional candidates. We found that the burden of amino acid changes in *IL18R1* and *BCL10* was significantly different between leprosy cases and healthy controls, suggesting these genes as functional candidates in leprosy pathogenesis.

## Introduction

Leprosy and tuberculosis (TB) are the two most prevalent mycobacterial infectious diseases worldwide. Compared to TB, the number of active leprosy cases has drop considerably in the past decades. This was achieved mostly due to a combined effort of healthcare professionals, changes in government policies and the use of a free and effective multi-drug therapy [1]. Despite these efforts, leprosy incidence has seen little change in recent years. More than 200,000 new cases are reported yearly since 2010 [2]. Preventing active disease transmission remains a main focus of the World Health Organization towards leprosy eradication. Drug-resistant *Mycobacterium leprae* and different bacterial lineages have been reported, but they likely provide a minor contribution to the sustained leprosy incidence [3,4]. The existence of host genetic risk factors in leprosy has long been established [5]. Hence, the stability in leprosy incidence can, at least partially, be attributed to a high proportion of individuals who are genetically susceptible to *M*. *leprae* infection and subsequent disease.

Candidate gene approaches and fine mapping of linkage peaks have identified variants and loci contributing to leprosy susceptibility. But it was the advent of genome-wide association studies (GWAS) that substantially increased our understanding of the host genetic component in leprosy. Currently, more than 30 independent loci have been shown to be associated with leprosy *per se* [5]. A limitation of GWAS results is that reported loci commonly encompass multiple genes, which poses a challenge to define the functional candidates in each locus. Techniques such as linkage disequilibrium score regression, expression quantitative trait loci (eQTL) and co-localization analyses can narrow down the list of potential functional candidate genes but are not without their own limitations. Here, we hypothesized that genes located in GWAS loci present a significantly different burden of protein-altering variants between leprosy cases and healthy controls. In the vast majority, amino acid variants that impact protein function are rare and will be missed by association scans employing genotyped common variants. In addition, imputed rare variants usually have low imputation quality and do not pass quality control filters. Fine mapping GWAS loci via deep resequencing allows the identification of such rare or novel variants. Targeted deep resequencing has successfully identified rare protein altering variants associated with complex diseases including inflammatory bowel disease (IBD; [6]), Alzheimer disease [7], autoimmune thyroid disease [8], and cancer [9]. Using this approach, we observed a significant depletion of protein-altering variants in leprosy cases for the *IL18R1* and *BCL10* genes, identifying them as candidate genes for a protective role in leprosy pathogenesis. Similarly, we identified a suggestive signal for depletion of amino acid changes for the *CDSN* and *PSORS1C2* genes in leprosy cases, suggesting them as novel functional candidates for leprosy on chromosome 6p21.

## Methods

### Ethics statement

The study received approval from the regulatory authorities of Ho Chi Minh City, Vietnam (So3813/UB-VX and 4933/UBND-VX) and the Research Ethics Board of the Research Institute at McGill University Health Centre in Montreal, Canada (REC98-041). Written informed consent was obtained from all participants in the study. For children, assent was given by subjects, and written informed consent was obtained from parents or guardians.

### Population sample and deep resequencing

For the study, we selected 555 independent leprosy-affected cases and 500 controls from our records and banked DNA samples of the Vietnamese population *from Ho Chi Minh City metropolitan area* (Table 1). Controls were clinically healthy subjects at the time of enrolment with no history of leprosy, tuberculosis, metabolic disease, cancer or immune- mediated disease. Sex and age significantly differed between cases and controls. For the 1055 selected subjects, an Illumina TrueSeq Custom Amplicon v1.5 panel was used to target the transcribed region of 34 genes identified as candidates by leprosy GWAS or positional cloning (S1 Table). Genes suggested as functional candidates in the original articles published between 2004 and 2015 were selected for deep resequencing [10–17]. Additionally, three candidate genes described in a recent GWAS of the Vietnamese population were selected for deep sequencing [18].

**Table 1:** Studied population sample.

	Leprosy	Healthy
	cases	controls
N	555	500
**Age at diagnosis/recruitment**		
Mean (SD)	20.1 (5.4)	30.5 (8.3)
**Gender**		
Male	384 (69.2)	273 (54.6)
Female	171 (30.8)	227 (45.4)
**Ridley-Jopling Clinical subtype**		
TT	14 (2.5)	-
BT	125 (22.5)	-
BB	221 (39.8)	-
BL	182 (32.8)	-
LL	13 (2.4)	-
**Leprosy type-1 reaction**		
Yes	239 (43.1)	-
No	316 (56.9)	-

Of the 1055 selected samples, 24 failed library preparation. The remaining 1031 samples were paired-end sequenced with Illumina MiSeq 600 cycles kit v3 in MiSeq. The acquired sequences were aligned to the human genome (hg19) using a banded Smith-Waterman algorithm from Illumina and variant calling was performed with the GATK v3 pipeline [19]. Quality control of variants was carried out using the same parameters as described previously [20]. Of the 1031 samples sequenced, three presented excess of heterozygotes and were excluded from the analyses. The 1028 samples that passed quality control had a mean depth of coverage of 214X (95% confidence interval ranging from 35X to 392X). ANNOVAR was used to annotate variant function [21].

### Statistical analyses

Genes that presented more than three protein-altering variants were selected for the gene-wise analysis. We have previously shown that amino acid variants predicted not to modulate functional activity of a protein had measurable effects on protein function [20]. Therefore, our analyses included all protein-altering variants. The enrichment or depletion of protein-altering variants per gene was tested using the optimal unified burden and SNP-set Kernel Association Test (SKAT-O) adjusting for small sample size as implemented in the software EPACTS [22,23]. Bonferroni was used for multiple testing correction considering 24 tested genes with more than three protein-altering variants resulting in a significance cut-off of *P* < 0.002. Transcribed SNPs with a minor allele frequency (MAF) > 0.01 were tested in a univariable additive model for their association with leprosy using PLINK 1.9 [24]. For the genes that were significant in the gene-wise analysis and had a protein-altering variant with *P* < 0.05 in the univariable model, we performed conditional analysis. Briefly, the variant nominally significant at the univariable model was censored in the SKAT-O variant count matrix and included as a covariate in the SKAT-O modeling. The SKAT-O and both the conditional and univariable analysis included sex as a covariate. Healthy controls were on average 10 years older than leprosy cases. Age was not included as a covariate as the control group had more time to be exposed and develop leprosy and therefore age would not be a confounder in our analysis. To test the combined effect of *IL18R1* rare protein-altering variants identified by our study with those described for the Chinese population [25,26], we used a Cohort Allelic Sums Test (CAST) implemented in AssotesteR [27].

### Databases

Linkage disequilibrium (LD) pattern between variants described by our study and common variants not genotyped in our samples were compared to the East Asian (EAS) population of the 1000 genomes in Ensembl [28]. LD between variants identified in our population as well as with the HLA alleles was estimated with PLINK 1.9 and snp.plotter [24,29]. The predicted deleterious effect of amino acid changes reported by our study was extracted from five databases (SIFT, PolyPhen2 HDIV, PolyPhen2 HVAR, LRT and Mutation tester) with the CADD score reported separately (S2 Table).

### Spatial modeling of IL18R1

To visualize the spatial localization of mutations we used a crystallography model of the heterodimer complex of IL18R1 with IL18RAP (Protein Data Bank, PDB:3WO3) [30]. The structural impact of amino acid substitutions in IL18R1 was predicted with PyMol [31]. Briefly, rearrangements in IL18R1 conformation were estimated for amino acids in a five Armstrong (Å) radius from the most likely rotamer of the mutated residuals. The impact of polarity and hydrogen bonding was also assessed for amino acids surrounding the mutated site. The impact of IL18R1 variants located in the TIR (Toll/interleukin-1 receptor) domain could not be assessed as the crystallography data included only the extracellular complex of the IL18 receptors.

## Results

Of the 34 genes targeted for deep resequencing, *RAB32* and *SOCS1* failed library preparation (S1 Table). For the remaining 32 genes, we identified 770 transcribed variants, of which 234 resulted in altered protein amino acid sequences (S2 Table). Three genes (*TNFSF8*, *BATF3*, and *ADO*) had no protein-altering variants and five genes (*TNF*, *IL12B*, *RMI2*, *DEC1* and *PSORS1C1*) had three or less in the studied sample, thus, were not evaluated in the gene-wise analysis (S1 Table). When testing the remaining 24 genes for enrichment or depletion of protein-altering variants in leprosy patients compared to healthy controls, the *IL18R1* and the *BCL10* genes displayed significant evidence for association with leprosy surpassing the multiple testing correction cut-off of *P* < 0.002 (Table 2). Furthermore, mutations in the *CDSN* and *PSORS1C2* genes located at 1.2kb and 25kb, respectively, from the top SNP (rs1265048) of the novel GWAS hit in the Vietnamese population showed a trend for protection from leprosy. By contrast, none of the remaining genes showed nominal significant evidence of association with leprosy (Table 2).

**Table 2:** Gene-wise analysis of protein-altering variants.

Targeted gene	Number of protein-altering variants		SKAT-O (*P*-value)^*b*^
	Total	Singleton^*a*^	
*PRKN*	15	10	0.24
*PACRG*	4	2	0.56
*LTA*	5	1	0.55
*LACC1*	5	3	0.29
*CCDC122*	4	3	0.21
*RIPK2*	8	5	0.60
*NOD2*	17	10	0.16
*SNX20*	6	4	0.66
*LRRK2*	25	14	0.59
*TNFSF15*	4	2	0.06
*TLR1*	18	9	0.46
*IL23R*	12	5	0.08
*IL12RB2*	15	11	0.31
*IL1RL1*	13	4	0.09
*IL18R1*	6	3	0.0004 ^*c*^
*IL18RAP*	9	6	0.43
*BCL10*	4	3	0.0019 ^*c*^
*CDH18*	5	4	0.64
*EGR2*	4	4	0.38
*CCDC88B*	9	4	0.63
*COX4I1*	8	4	0.23
*SIGLEC5*	8	2	0.59
*CDSN*	14	5	0.006
*PSORS1C2*	4	1	0.02
*IL18R1/IL1RL1*	19	7	0.004
*IL18R1/IL18RAP*	15	9	0.004
*IL18R1/IL1RL1/IL18RAP*	28	13	0.003

^*a*^ Singletons are rare variants observed as heterozygotes in a single individual of the studied population.

^*b*^ SKAT-O *P*-value estimate the significant enrichment or depletion of protein-altering variants in either leprosy cases or healthy controls.

^*c*^ Protein-altering variants were significantly more frequent in healthy controls.

**Fig 1:**
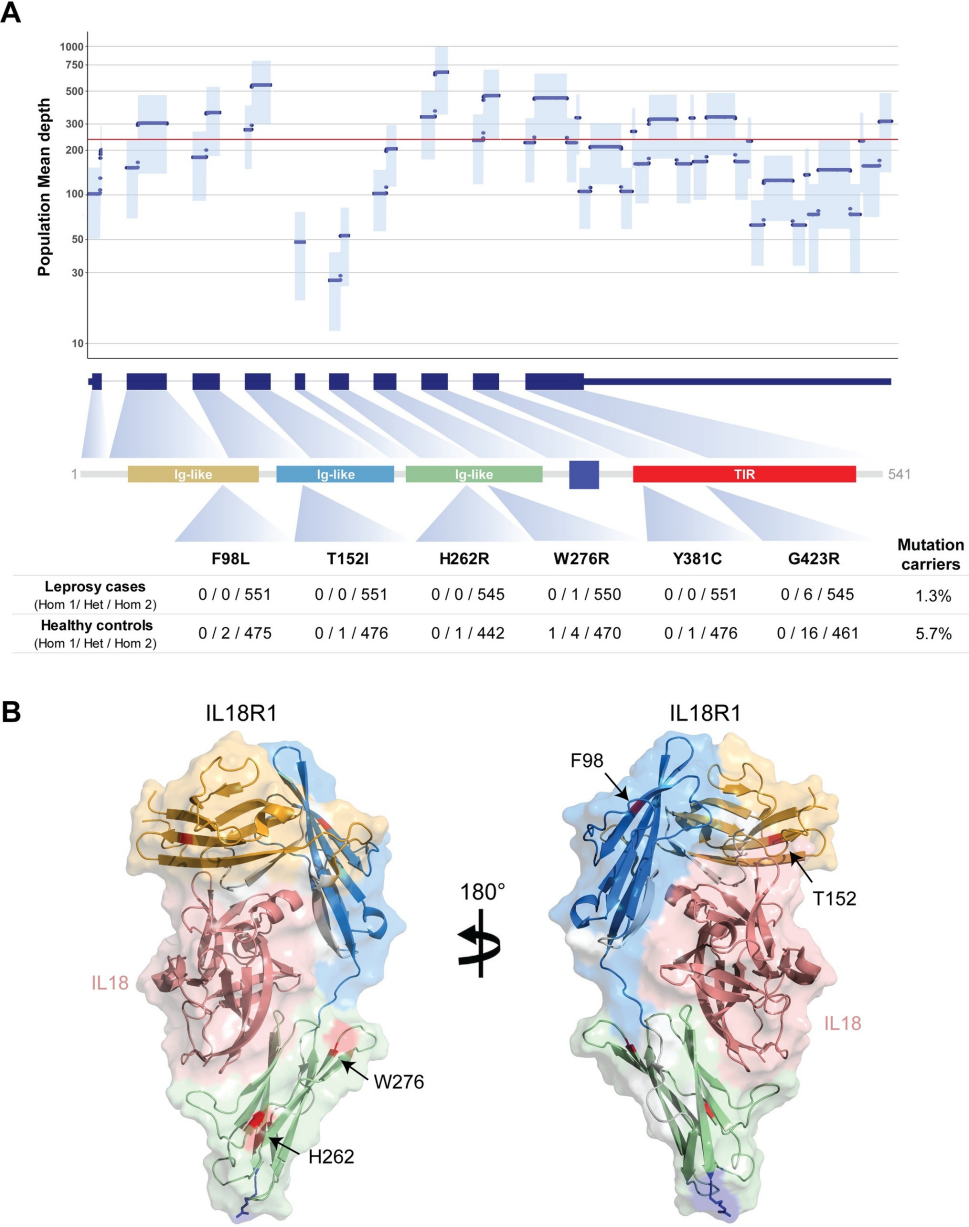
Depletion of IL18R1 amino acid changes in leprosy cases. (**A**) At the top, the population mean-depth of coverage is plotted according to the exons encoding the *IL18R1* gene. Dark blue dots denote the depth of coverage with the light blue shade indicating one standard deviation of the mean. A red horizontal line marks the average depth of coverage for the *IL18R1* locus in the studied population. The protein domains are shown in the center linked to their respective encoding exons (dark blue boxes below the plot). At the bottom, the allele counts and the proportion of mutation carriers for the *IL18R1* gene are shown for leprosy cases and healthy controls. Hom 1: homozygous for the reference allele; Het: heterozygous; Hom 2: homozygous for the mutation. (**B**) Crystallographic model for the extracellular domains of IL18R1 coupled with IL18 (PDB:3WO3). The mutated residues observed in the present study are highlighted in red and indicated by arrows. The IL18R1 protein contains three extracellular Ig-like domains and an intracellular signaling domain TIR (Toll/interleukin-1 receptor). Four out of the six IL18R1 amino acid changes identified in our study are located in beta-sheets of the extracellular Ig-like domain, while two mutations altered the TIR signaling domain. The crystallographic model of IL18 receptors does not include the intracellular TIR domain, which are responsible for signaling the transduction of IL1/IL18 and TLRs receptors by interacting with MyD88 and TOLLIP to activate the signaling cascade. The IL18R1 p.G423R amino acid change is located in a conserved region of the TIR domain and the substitution from the backbone amino acid Glycine to a polar Arginine is predicted to be deleterious in three of the five functional databases curated.

For the *IL18R1* gene, we observed six rare or low-frequency amino acid changes in the studied sample (Fig 1A). A total of 5.7% (26/477) of the healthy subjects carried at least one missense allele while only 1.3% (7/551) of the leprosy cases displayed a nonsynonymous *IL18R1* variant (Fig 1A). None of the studied subjects carried more than one *IL18R1* missense mutation. Combining *IL18R1* with the other genes in the 2q12.1 GWAS locus–*IL18RAP*, *IL1RL1* or both–did not improve the gene-wise association of *IL18R1* with leprosy (Table 2). This observation identifies *IL18R1* as the functional candidate of leprosy susceptibility regarding protein-altering variants. The most frequent IL18R1 missense variant (p.G423R) is located in the TIR (Toll/interleukin-1 receptor) domain while four of the rare variants are located in the extracellular complex of IL18R1 formed by three Ig-like domains (Figs 1B and S1). Spatial modeling of variants in the extracellular domains of IL18R1 showed incompatibility between the alternative amino acid and the surrounding alpha helixes and beta sheets. The insertion of alternative amino acids created a structural change that was expected to force a reshaping of the domain and impact the affinity of IL18 to its receptor (S1 Fig). The p.G423R mutation was a low-frequency variant observed with a frequency of 1.7% in healthy controls and 0.5% in leprosy cases. In a univariable model, the R allele for p.G423R was nominally associated with leprosy protection (*P* = 0.015, S2 Table). We conditioned the gene-wise association of *IL18R1* for the p.G423R and observed that the additional amino acid changes remained nominally significantly depleted in the gene (*P*_*SKAT-O*_ = 0.02) indicating that an increasing burden of nonsynonymous variants in *IL18R1* is protective for leprosy independent of the p.G423R mutation. Rare amino acid changes in *IL18R1* had also been identified in a case-control study of Chinese leprosy patients [25,26]. When we combined *IL18R1* rare variants (MAF < 1%) detected in the Chinese and the Vietnamese population samples, significant depletion was maintained for rare variants in leprosy cases (*P*_CAST_ = 0.001) surpassing multiple test correction (Table 3). Taken together, these results directly implicated amino acid changes in IL18R1 and not its genomic neighbors as leprosy protective factors.

**Table 3:** Depletion of IL18R1 rare amino acid changes in leprosy cases of Asian descent.

Population	IL18R1 variant	Leprosy cases (Alt/Ref allele) counts	Healthy controls (Alt/Ref alleles) count	CAST (*P*-value)
Chinese	p.F97V	0/1596	1/1980	
	p.N110S	0/1596	1/1978	
	p.R210H	0/1596	1/1980	
	p.Y381C	0/1596	1/1980	
	p.G423R	42/1596	44/1980	
	p.M520L	0/1596	1/1980	
Vietnamese	p.F98L	0/1102	2/954	
	p.T152I	0/1102	1/954	
	p.H262R	0/1090	1/880	
	p.W276R	1/1102	5/944	
	p.Y381C	0/1102	1/954	
	p.G423R	6/1102	16/954	
**Count** (MAF < 5%)		49/16176	75/17518	0.08
**Count** (MAF < 1%)		1/13478	16/14584	0.001

MAF; minor allele frequency. Alt; Alternative allele. Ref; Reference allele. CAST estimate differences in the cumulative allelic frequency between cases and controls by combining listed variants in a “super variant”. The bottom two rows show the cumulative allelic count for variants with MAF < 5% or MAF < 1% used as threshold for inclusion in CAST analysis.

The gene-wise association of *BCL10* with leprosy was mainly driven by the common p.G213E variant. In a univariable model, the E allele of the p.G213E variant was associated with leprosy protection (*P* = 0.01, S2 Table). The *BCL10* gene-wise association with leprosy lost significance when conditioning for the p.G213E variant (*P*_*SKAT-O*_ = 0.08). In the Vietnamese population, the p.G213E is independent (*r*^2^ = 0.075) of the common variant rs2735591 identified by GWAS in Chinese leprosy patients [16].

A GWAS and HLA fine-mapping strategy in the Vietnamese population identified rs1265048 located at 1.2kb of the *CDSN*/*PSORS1C1* genes and 25kb from the *PSORS1C2* gene in the MHC region as being independent from classical HLA alleles associated with leprosy. Using the gene-wise burden of amino acid changes, we identified a trend for the *CDSN* and *PSORS1C2* genes as functional candidates for leprosy. The signal for the two genes was captured mostly by two low frequency variants (MAF ~ 0.025) in *CDSN* p.G36S and the frameshift *PSORS1C2* p.P94fs that are in high linkage disequilibrium (LD; *r*^2^ = 0.87 in healthy controls and *r*^2^ = 0.70 in leprosy cases; S2 Fig). Both mutations were a significant protective factor for leprosy in the univariable model (*P*~0.01; S2 Table). Conditioning the gene-wise analysis for the p.G36S, p.P94fs mutations or HLA-C*07:*06* (allele with LD *r*^2^ between 0.7 and 1 with the two mutations in both cases and controls) the gene-wise association of *CDSN* or *PSORS1C2* with leprosy lost significance (*P*_*SKAT-O*_ ranging from 0.08 to 0.47). In our conditioning analysis we were unable to dissociate the protection signal for *CDSN*/*PSORS1C2* amino acid changes from our previously reported HLA-C*07:*06* association with leprosy. Moreover, the low frequency amino acid changes in *CDSN* and *PSORS1C2* were not tagged by the common leading variant rs1265048 (MAF = 0.49) described by our GWAS (*r* ^2^ = 0.01). This observation suggests that the leading SNP in the GWAS captured a signal independent from the low frequency variants in *CDSN*, PSORS1C2 and HLA-C*07:*06*.

## Discussion

Leprosy stands out among infectious diseases as an example where susceptibility to disease is strongly dependent on the host genetic background. Here we studied the contribution of rare amino acid mutations by analysing the gene-wise burden of protein-altering variants. We identified mutations in four genes (*IL18R1*, *BCL10*, *CDSN*, and *PSORS1C2*) as candidates for functional mediators of leprosy susceptibility. BCL10 is a CARD domain carrying protein that can trigger apoptosis and activate NF-κB. SNPs in vicinity of the *BCL10* gene were associated with leprosy *per se* in a large sample of Chinese leprosy patients [16]. A role of the *BCL10* gene in leprosy susceptibility is supported by: i) the biological function of the BCL10, ii) SNPs associated with leprosy are eQTLs for *BCL10* that may affect binding of the transcriptional factors FOXA1 and FOXA2 to *BCL10*, and iii) the lower expression of *BCL10* in the skin of leprosy patients compared to healthy controls [16]. However, the most statistically significant SNP rs233100 associated with leprosy is an eQTL for a long non-coding RNA (*RP11-131L23*.*1*) raising the possibility that the genetic effect may be mediated independently of *BCL10*. Here, the identification of the BCL10 p.G213E variant being significantly depleted in leprosy cases provided independent support for the role of *BCL10* in leprosy pathogenesis. Interestingly, when the univariable analysis was stratified by sex the association of p.G213E was significant only in the female group (*P* = 0.002) and not in males (*P* = 0.32). This observation suggests that some variants may contribute to the sex bias on leprosy susceptibility, an area of research that remains poorly explored.

A recent GWAS in the Vietnamese population identified three independent association signals for leprosy in the MHC region [18]. Two of these signals tagged classical HLA alleles identified by HLA sequencing [32], while the third signal located near a major risk locus for psoriasis that encompassed *CDSN*, *PSORS1C1*/*PSORS1C2* and *C6orf15* [18,33]. Here, we identified two low-frequency variants in high LD (p.G36S–p.P94fs) altering both *CDSN*–*PSORS1C2* as being protective from leprosy independent of the GWAS leading SNP [18]. A common functional synonymous mutation Y319Y in *CDSN* had previously been associated with psoriasis independently of *HLA-C* alleles [34,35]. HLA fine mapping showed the HLA-C*07:*06* allele to be associated with leprosy protection with a similar frequency and the same direction of association as the *CDSN*–*PSORS1C2* amino acid changes [32]. This led us to evaluate if the *CDSN* and *PSORS1C2* association with leprosy protection was independent of the HLA-C*07:*06* allele. The conditional analysis could not distinguish the association of CDSN/PSORS1C2 with leprosy from the HLA-C*07:06 protective effect. The HLA-C gene is a clear functional candidate, however, CDSN has also been shown as a promising candidate for immune mediated diseases. For example, Cormeodesmosin (encoded by *CDSN*) is overexpressed in psoriatic lesions and has been suggested to contribute to skin desquamation [36,37]. Moreover, the CDSN p.S453N variant is associated with Crohn’s disease (CD) in the Ashkenazi Jewish population [38]. A host genetic overlap between CD and leprosy is well established [39], while psoriasis affects mostly skin tissue as observed in leprosy. The association of *CDSN* with psoriasis, CD and now leprosy suggests CDSN as an attractive functional candidate for further exploration in leprosy.

Our results are of particular interest for the role of IL18 signalling. IL18 is a prototypical pro-inflammatory cytokine best known for its ability to induce IFNγ [40]. IL18 is mainly secreted by dendritic cells and macrophages and its signaling is mediated through the IL18 receptor. The IL18 receptor is a heterodimeric molecule of IL18R-alpha (encoded by *IL18R1*) and IL18R-beta (encoded by *IL18RAP*). IL18 binds primarily to IL18R-alpha while signaling is mediated through the IL18R-beta chain. The IL18 receptor is mostly expressed on hematopoietic cells, including CD4+ T cells, NK cells and mast cells. The leading variant for the 2q12.1 GWAS leprosy susceptibility locus, rs2058660, had been associated with protection from CD in an extension of the well-established overlap of leprosy host genetic factors with genetic modulators of CD [15,41]. Opposite directions of associations for leprosy and CD are consistent with the suggestion that effective pro-inflammatory host responses protect from infectious disease but predispose to inflammatory disorders like CD. However, rs2058660 is an eQTL for *IL18RAP* in neutrophils where the allele associated with leprosy risk (and CD protection) drives lower expression of *IL18RAP*, hence a more subdued pro-inflammatory signalling by IL18 [42]. The role of IL18 in maintaining gut mucosal homeostasis and tissue integrity during CD is a matter of controversy. On the one hand, studies with knock-out mice suggested a critical role of IL18 in protection from intestinal inflammation and colitis while on the other hand IL18 was shown to promote goblet cell dysfunction and breakdown of the mucosal barrier [43,44]. In either case, it seems likely that the role of IL18 in inflammatory bowel disease and leprosy involve different aspects of IL18 function.

The effects of eQTLs on gene expression are cell-type specific and often implicate more than a single gene. For example, the leprosy susceptibility allele of rs2058660 is associated with reduced *IL18RAP* expression in blood but increased expression of *IL18R1* in the lung [45]. The apparent contradiction between effect on gene expression and genetic associations of rs2058660, might have been caused by focusing on the eQTL effect for the wrong gene and or the wrong cell type. Our results strongly argue against this possibility since loss of function mutations in *IL18R1* are depleted in leprosy cases supporting the conclusion from the eQTL finding that reduced IL18 signalling is protective for leprosy. It is therefore noteworthy that IL18 signalling does not necessarily trigger a pro-inflammatory response. Rather, the pro-inflammatory IL18 signalling is dependent on the presence of IL12 [46]. It has been shown that in the absence of IL12, IL18 can trigger an anti-inflammatory response [46]. Since IL18 can be produced by variety of cells, including non-immune cells, this suggests that IL18 production is triggered at early stages of leprosy and CD before the onset of acquired immunity and the initiation of the IL12-IFNγ loop. At this stage of pathogenesis, blunted IL18 signalling in leprosy either by reduced *IL18RAP* expression or reduced IL18 binding by IL18R1 would hinder the development of anti-inflammatory host responses, which is expected to be beneficial for countering an infectious agent. The subsequent IL12-dependent pro-inflammatory effects of IL18 appear redundant in the presence of an emerging anti-*M*. *leprae* host response.

Our study also has limitations. For example, the PCR-based protocol used for deep resequencing precluded the identification of large structural variants and CNVs that could impact protein structure and function. Moreover, the lack of replication for the IL18R1 p.G423R mutation in the Chinese population was unexpected. It is possible that other functional low frequency variants not evaluated by our study are in LD with p.G423R only in the Vietnamese population. In this case, the p.G423R variant would be a tag SNP. We think that this is an unlikely scenario as p.G423R is an excellent candidate. The mutation was predicted to be deleterious in multiple databases and had one of the highest CADD scores in our study (S2 Table). Likewise, while IL18R1 rare amino acid changes are good functional candidates, we cannot exclude the possibility of LD between protein altering variants and other rare tagging SNPs. While the depletion of rare amino acid changes for IL18R1 was detected for Vietnamese and Chinese leprosy cases [26], the reason for this depletion remains unknown. Studies with larger sample size evaluating long range LD across the IL18R1 locus or assessing the impact of IL18R1 amino acid changes on host responses to M. leprae using cellular models, as applied in [20,47], will be necessary to fully dissect the contribution of IL18R1 to leprosy pathogenesis. In addition, we cannot exclude the possibility of un-captured phenotypic heterogeneity in the control group and possible pleiotropic effects of the variants found preferentially in this group.

## Supporting information

S1 Fig Amino acid changes observed in the extracellular domain of IL18R1. Zoom in view at the IL18R1 mutated residues detected in the present study. The reference amino acid is shown at the top with the best fitting isomer for the alternative amino acid shown at the bottom.
(TIF)

S2 Fig Linkage disequilibrium between CDSN/PSORS1C2 amino acid changes and HLA alleles. The diamond plots show the linkage disequilibrium *r*^2^ for 977 individuals separately for 531 leprosy cases and 439 healthy controls.
(PDF)

S1 Table Genes targeted for deep resequencing in leprosy *per se*. (XLSX)

S2 Table Summary of the 770 transcribed variants in the targeted genes. (XLSX)
