# Supplementary figures and images for: Deep resequencing identifies candidate functional genes in leprosy GWAS loci
Source: PLoS Negl Trop Dis. 2021 Dec 8;15(12):e0010029. doi: 10.1371/journal.pntd.0010029 (PMC8687567; doi:10.1371/journal.pntd.0010029)

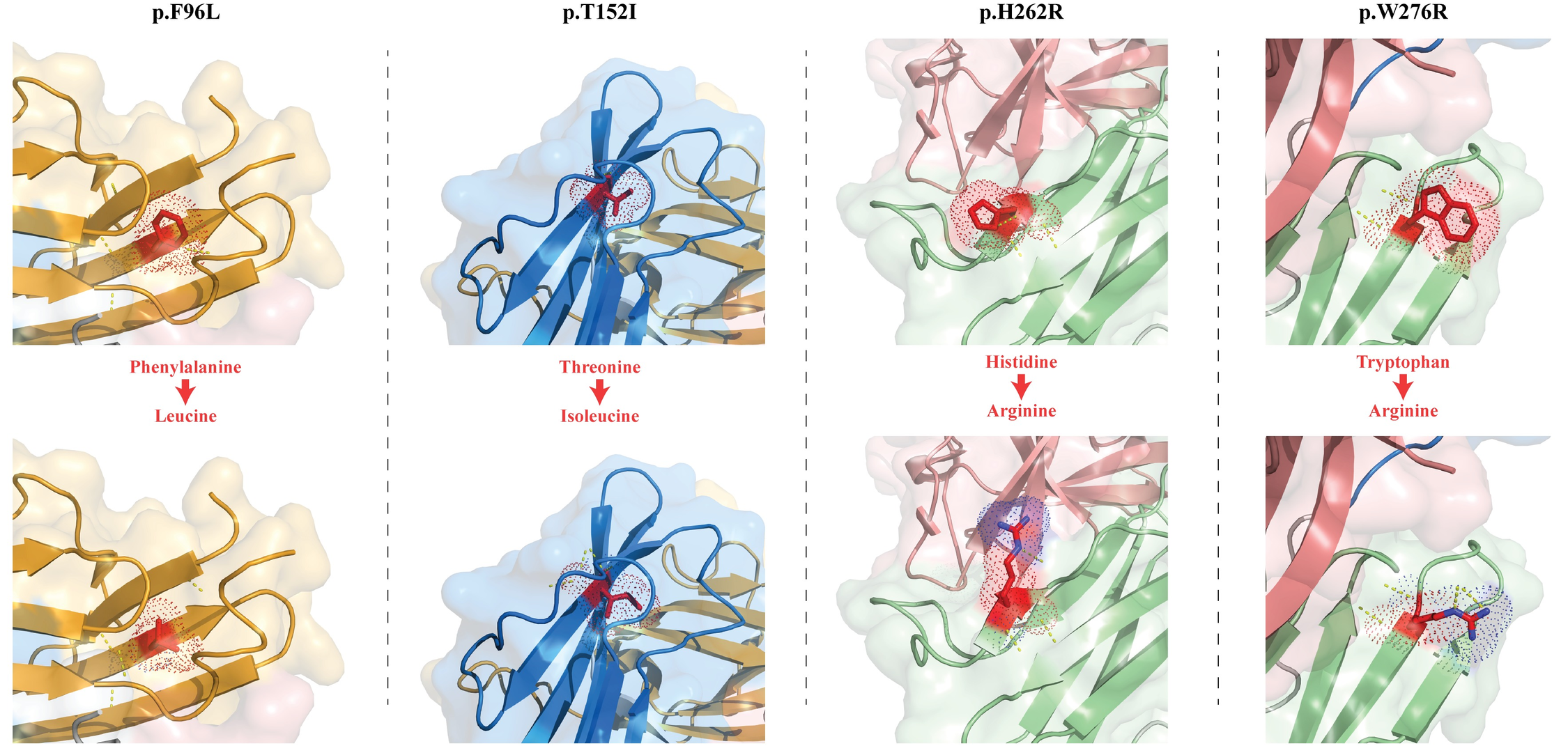

Supplement: S1 Fig — Zoom in view at the IL18R1 mutated residues detected in the present study. The reference amino acid is shown at the top with the best fitting isomer for the alternative amino acid shown at the bottom. (TIF) [file pntd.0010029.s001.tif]

439

Healthy controls

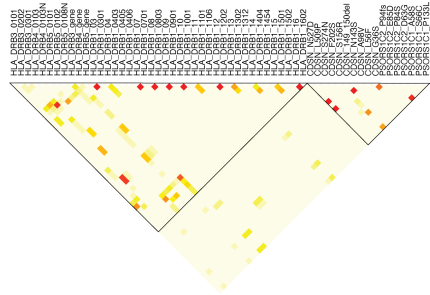

531

Leprosy cases

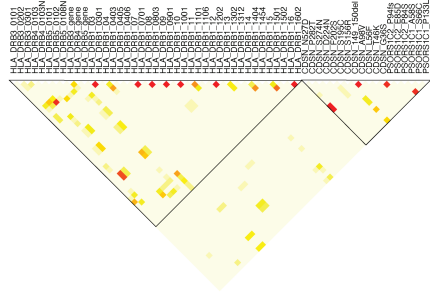

439

Healthy controls

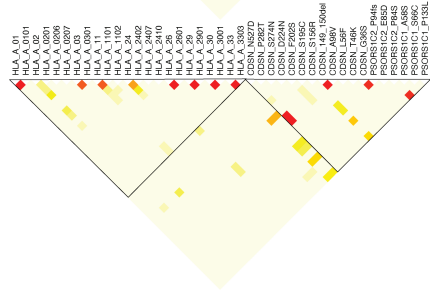

531

Leprosy cases

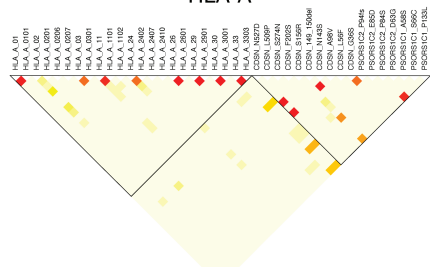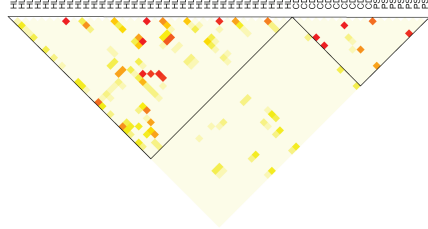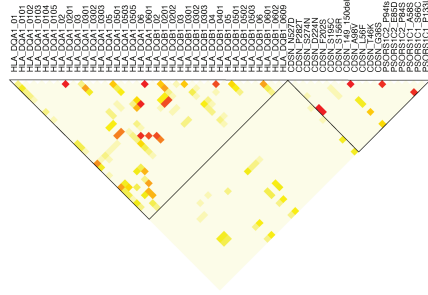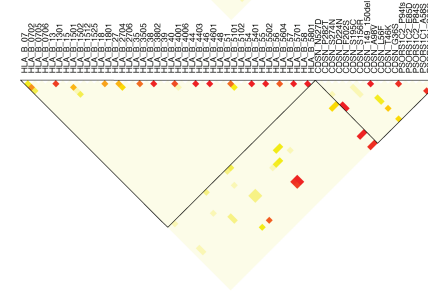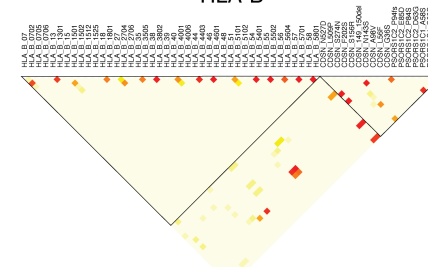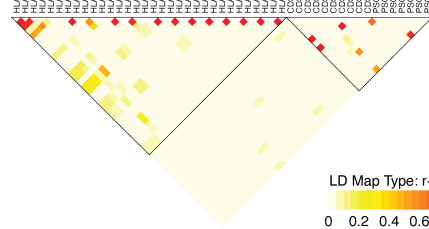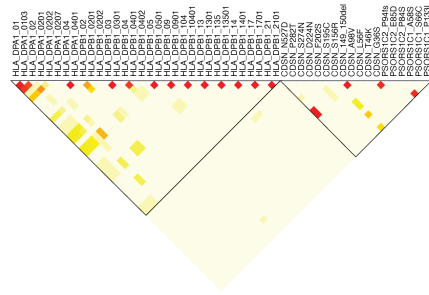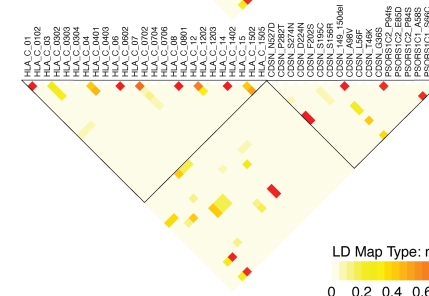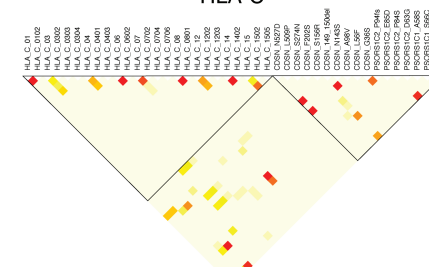

Supplement: S2 Fig — The diamond plots show the linkage disequilibrium r2 for 977 individuals separately for 531 leprosy cases and 439 healthy controls. (PDF) [file pntd.0010029.s002.pdf]
